# Risk factors associated with adverse maternal outcomes following intrapartum cesarean birth: a secondary analysis of the WHO global survey on maternal and perinatal health, 2004–2008

**DOI:** 10.1186/s12884-020-03390-0

**Published:** 2020-11-11

**Authors:** Margo S. Harrison, Ana Pilar Betrán, Krithika Suresh, Joshua P. Vogel, Robert L. Goldenberg, A. Metin Gülmezoglu

**Affiliations:** 1 University of Colorado School of Medicine, Mail Stop B198-2, Academic Office 1, 12631 E. 17th Avenue, Rm 4211, Aurora, CO 80045 USA; 2 UNDP/UNFPA/UNICEF/WHO/World Bank Special Programme of Research, Development and Research Training in Human Reproduction, Department of Sexual and Reproductive Health and Research, World Health Organization, Geneva, Switzerland; 3 Department of Biostatistics and Informatics, University of Colorado, Aurora, CO USA; 4 Maternal, Child and Adolescent Health Program, Burnet Institute, Melbourne, Australia; 5 Department of Obstetrics and Gynecology, Columbia University Medical Center, New York, NY USA

**Keywords:** Cesarean birth, Unplanned, Intrapartum, Low- and middle-income countries

## Abstract

**Background:**

To identify risk factors associated with a composite adverse maternal outcomes in women undergoing intrapartum cesarean birth.

**Methods:**

We used the facility-based, multi-country, cross-sectional WHO Global Survey of Maternal and Perinatal Health (2004–2008) to examine associations between woman-, labor/obstetric-, and facility-level characteristics and a composite adverse maternal outcome of postpartum morbidity and mortality. This analysis was performed among women who underwent intrapartum cesarean birth during the course of labor.

**Results:**

We analyzed outcomes of 29,516 women from low- and middle-income countries who underwent intrapartum cesarean birth between the gestational ages of 24 and 43 weeks, 3.5% (1040) of whom experienced the composite adverse maternal outcome. In adjusted analyses, factors associated with a decreased risk of the adverse maternal outcome associated with intrapartum cesarean birth included having four or more antenatal visits (AOR 0.60; 95% CI: 0.43–0.84; *p* = 0.003), delivering in a medium- or high-human development index country (vs. low-human development index country: AOR 0.07; 95% CI: 0.01–0.85 and AOR 0.02; 95% CI: 0.001–0.39, respectively; *p* = 0.03), and malpresentation (vs. cephalic: breech AOR 0.52; CI: 0.31–0.87; *p* = 0.04). Women who were medically high risk (vs. not medically high risk: AOR 1.81; CI: 1.30–2.51, *p* < 0.0004), had less education (0–6 years) (vs. 13+ years; AOR 1.64; CI: 1.03–2.63; *p* = 0.01), were obstetrically high risk (vs. not high risk; AOR 3.67; CI: 2.58–5.23; *p* < 0.0001), or had a maternal or obstetric indication (vs. elective: AOR 4.74; CI: 2.36–9.50; *p* < 0.0001) had increased odds of the adverse outcome.

**Conclusion:**

We found reduced adverse maternal outcomes of intrapartum cesarean birth in women with ≥ 4 antenatal visits, those who delivered in a medium or high human development index country, and those with malpresenting fetuses. Maternal adverse outcomes associated with intrapartum cesarean birth were medically and obstetrically high risk women, those with less education, and those with a maternal or obstetric indication for intrapartum cesarean birth.

## Key message

Certain woman-level, obstetric-level, and available facility-level risk factors are associated with a greater likelihood of adverse maternal outcomes following an intrapartum cesarean birth. The modifiable factors could be targeted for interventions to reduce adverse maternal outcomes of intrapartum cesarean birth.

## Background

Cesarean birth rates are increasing gobally [1]. This increase in cesarean birth rates is due, in part, to the performance of medically unnecessary cesareans [2]. Cesarean birth can be a life-saving procedure for mothers and babies, but it can also be associated with maternal morbidity and mortality [3]. Cesarean birth can potentially result in longer hospitalization and neonatal respiratory complications [4]. Compared with cesarean births conducted before the onset of labor, women are at the greatest risk of harm from cesarean birth when it is performed during labor, which is variably described as an unplanned, intrapartum, or emergency cesarean birth [5]. This is often due to lack of availability of anesthetic and surgical workforce and availability of supplies such as oxygen, anesthesia, and bloodbanks [6]. In order to mitigate the risks of adverse outcomes, cesarean birth should be used at the right time, for the right indications, and with appropriate surgical technique [7].

Given that cesarean birth rates are increasing globally, identifying actionable, modifiable risk factors associated with adverse maternal outcomes following intrapartum cesarean birth may help prevent some maternal morbidity and mortality related to this procedure [5]. We conducted a secondary analysis of the WHO Global Survey on Maternal and Perinatal Health dataset in order to compare women from low- and middle-income countries who gave birth by intrapartum cesarean and experienced an adverse maternal outcome, to women who experienced an intrapartum cesarean birth without having an adverse outcome [8]. Our aim was to identify any modifiable risk factors associated with a composite adverse maternal outcome following intrapartum cesarean birth to determine if there are any target areas that might improve pregnancy outcomes in this population.

## Methods

### Dataset

The methodology of the WHO Global Survey of Maternal and Perinatal Health (WHOGS) has been published [9]. In brief, WHOGS was undertaken in 2004–05 (in 8 Latin American and 7 African countries) and in 2007–08 in 9 Asian countries [10–12]. Data were gathered for 2 months in these low- and middle-income countries in institutions with at least 6000 deliveries per year and for 3 months in institutions with fewer than 6000 annual deliveries [9]. Data about the sociodemographic, obstetric, birth, and labor characteristics, and a range of maternal and perinatal outcomes, were captured from all women who gave birth in participating institutions during the data collection period [9]. Data were collected for 290,610 deliveries in 373 facilities in 24 countries [9]. Data were collected prospectively from the time of maternal presentation at the facility until discharge, death or the seventh day postpartum, whichever occurred first [9]. Data collectors reviewed medical records daily and abstracted de-identified data from these records into an individual data form [9]. Additionally, an institutional data form was completed for each participating facility via an interview with the head of the obstetrics/gynaecology department [9]. All countries were included except for Angola, which was dropped due to outlier data related to the ICU admission variable, which has been noted in prior WHOGS analyses [13].

### Study overview

This was a secondary analysis of the prospectively collected WHOGS data. Our study population included women who underwent intrapartum cesarean birth after the onset of spontaneous or induced labor between the gestational ages of 24 and 43 weeks. We compared women who experienced a composite adverse maternal outcome to those women who did not experience the composite outcome.

### Primary outcome

Our primary outcome of interest was a composite measure of severe maternal morbidity and mortality. A woman was considered to have had this composite outcome associated with cesarean birth if she experienced any one or more of the following: hysterectomy, intensive care unit (ICU) admission greater than or equal to 2 days, or maternal death [14]. It should be noted that an assumption of our analysis was that the ICU admission occurred after birth, but there is no method to verify this in the dataset, which was the methodology used in our major WHOGS analyses.

### Analysis

Covariates considered in our analysis were sociodemographic characteristics (education, human development index (2008) of the country where woman gave birth, number of antenatal visits, medical risk level (defined below), age, marital status, body mass index, obstetric risk level (defined below), referral to a higher level of care during the course of labor, gestational age, and birthweight [15]. We also included parity, number of fetuses, fetal presentation, onset of labor, and history of prior cesarean birth [16, 17]. Facility-level covariates considered in the analysis were teaching facility status, total deliveries at facility per year, and location of facility (urban versus rural).

The WHOGS collected the indication for cesarean birth as a checklist of 21 non-mutually exclusive possibilities; more than one indication for cesarean could be assigned to each woman [8, 9]. We considered the indication for cesarean birth in our analysis by dividing the 21 indications into five mutually exclusive groups—women had to have one of the indications in a given group, and none of the indications under the definition of another indication group; these are listed in Table 1.

**Table 1:** Mutually exclusive classification system for indication for intrapartum cesarean birth

Indication Group		Description
**1**	Fetal	Fetal growth restriction, fetal Distress, “other fetal indication”
**2**	Failure to Progress/Dystocia	Cephalopelvic disproportion, dystocia, failure to progress, failed vacuum or forceps, failed induction of labor, post-term, and suspected or imminent uterine rupture
**3**	Maternal/Obstetric	Pre-eclampsia, eclampsia, third trimester vaginal bleeding, “other maternal indication”, “other obstetric indication”
**4**	Multiples/Malpresentation	Multiple gestation, non-vertex presenting fetus
**5**	Elective	Previous cesarean section, tubal ligation/sterilization, maternal request, previous repaired urogenital fistula
**6**	No Other Indication	Does not have any of the other indications specified

For this secondary analysis, women were categorized into the “high” maternal medical risk category if the survey reported they had HIV, chronic hypertension, cardiac or renal disease, respiratory disease, diabetes, malaria, anemia, urinary tract infection, genital ulcers, or condyloma. In addition, we defined obstetric risk level as “high” for women who experienced pregnancy-related hypertension, pre-eclampsia or eclampsia, or suspected fetal growth impairment. Though multiple gestation, non-cephalic presenting fetuses, and history of prior cesarean birth are considered high-risk issues, these are considered obstetric variables and are presented separately from those who experienced the aforementioned pregnancy complications that made them “obstetrically high risk” for the purposes of this analysis.

Due to the hierarchical structure of the data, mixed effects logistic regression analyses was performed with a random effect for country and for facility nested within country. Univariate and multivariable analyses were used to assess the association between the adverse maternal composite outcome of cesarean birth and the identified facility- and individual-level covariates. *P*-values and 95% confidence intervals (CI) were reported, and statistical analyses was conducted using SAS v.9.4 software.

## Results

### Primary outcome

**Fig. 1:**
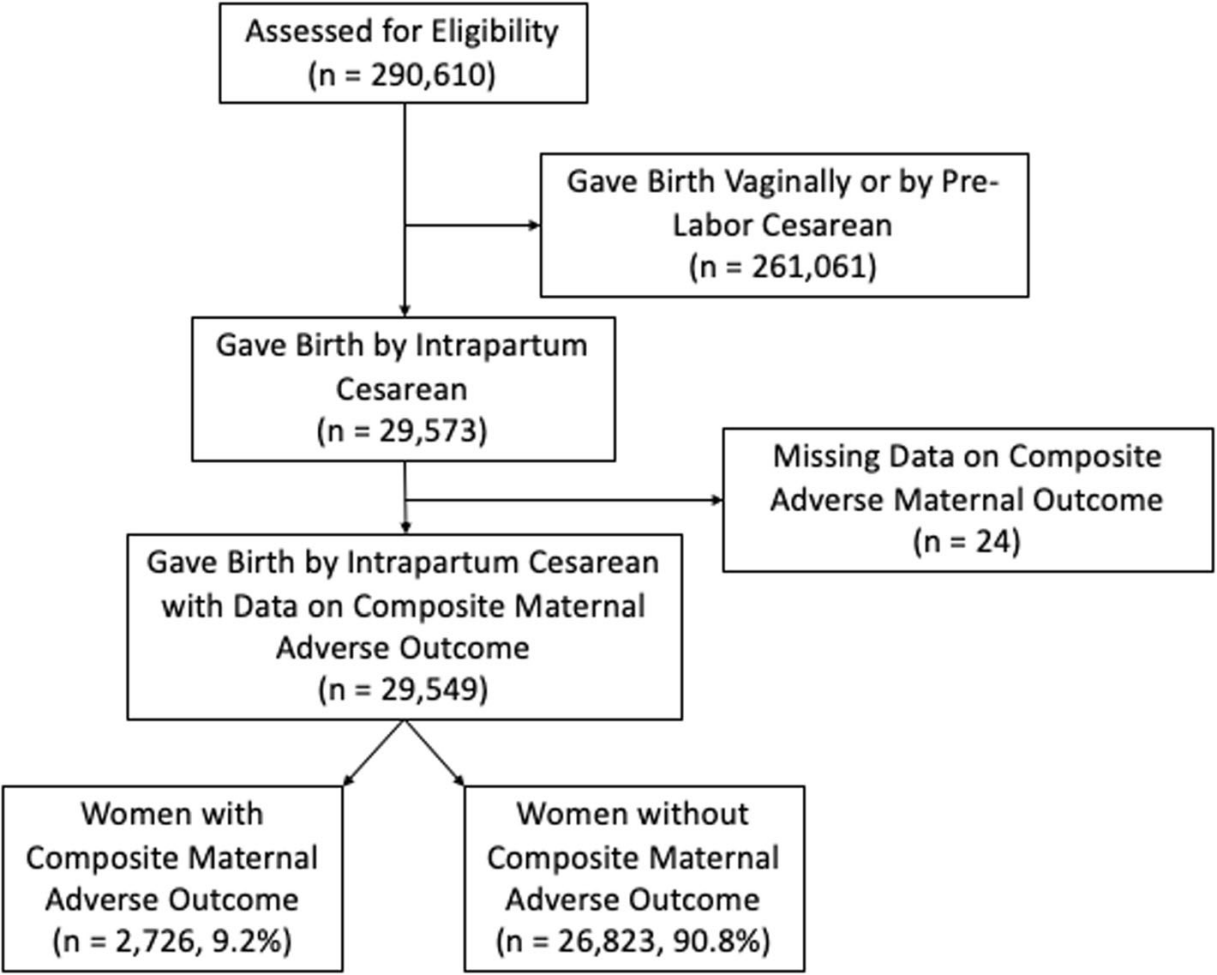
CONSORT diagram

From a total 290,610 women included in the WHOGS, 29,516 (10.2%) of women gave birth by intrapartum cesarean at a gestational age between 24 and 43 weeks and had the composite adverse outcome data available. Women experiencing cesarean birth prior to the onset of labor were not included in the analysis population. The CONSORT diagram in Fig. 1 illustrates the studied cohort. In total, 1040 (3.5%) of these women experienced the maternal adverse composite outcome (Table 2). The remaining 28,476 (96.5%) of the women experienced uncomplicated, intrapartum cesarean births.

**Table 2:** Prevalence of composite adverse maternal outcome

Complication	Prevalence (***n*** = 29,516)
Prevalence of Composite Outcome in Total Population	1040 (**3.5**%)
Prevalence Components of Composite:	
ICU Admission ≥ 2 Days	846 (**2.9%**)
Hysterectomy	160 (**0.5%**)
Maternal Death	49 (**0.2**%)

81.3% (846/1040) are from ICU admission, 15.4% (160/1040) are hysterectomy, 4.7% (49/1040) for death. These will sum to > 100% because people can fall into multiple categories

### Summary of populations

Table 3 summarizes the characteristics of the women in the study population. The majority of women lived in medium human development index countries (74%), attended four or more antenatal visits (72%), were not medically high risk (81%), were between the age of 19 and 34 (81%), had 7–12 years of education (55%), were married or cohabitating (87%), and were of normal (28%) or overweight (39%) body mass index. The majority of women had not had a cesarean (80%), were not obstetrically high-risk (90%), were not referred during the course of labor (74%), had a term birth (91%) of a cephalic fetus (87%), had a baby between 2500 and 3499 g (61%), went into spontaneous labor (86%), were nulliparous (53%), and delivered a singleton (97%) baby. Most women were delivered by an obstetrician (59%) at a teaching facility (82%), and at facilities that had 3500–9999 deliveries in a year (43%). 89% of deliveries were at facilities that were not in an urban setting. The most prevalent indication for cesarean birth was “no other indication” (32%), which was defined as not having another specific indication (Table 1), followed by failure to progress/dystocia (24%), fetal indication (17%), “elective” (11%), multiples or malpresentation (8%), and maternal or obstetric indication (7%).

**Table 3:** Summary of women-, obstetric-, labor-, and facility-level factors by adverse outcomes associated with intrapartum cesarean birth, WHOGS 2004–2008

	No Adverse Outcomes (***n*** = 28,476)		Adverse Outcomes (***n*** = 1040)		All (***n*** = 29,516)	
**Woman-Level Factors**						
**Human Development Index 2008 of Country in Which Woman Delivered**						
Low^a^	1394	5%	164	16%	1558	5%
Medium^b^	20,977	74%	801	77%	21,778	74%
High^c^	6105	21%	75	7%	6180	21%
**Antenatal Visits**						
< 4 visits	6211	22%	350	34%	6561	22%
4+ visits	20,489	72%	625	60%	21,114	72%
*Missing*	1776	6%	65	6%	1841	6%
**Medically high risk**^**d**^						
No	22,803	80%	2370	813	25,173	81%
Yes	5283	19%	342	217	5625	18%
*Missing*	*390*	*1%*	*10*	*0%*	*400*	*1%*
**Age groups**						
0–18	2016	7%	71	7%	2087	7%
19–34	23,210	82%	771	74%	23,981	81%
35+	3222	11%	189	18%	3411	12%
*Missing*	*28*	*0%*	*9*	*1%*	*37*	*0%*
**Education groups**						
0–6 years	6065	21%	308	30%	6373	22%
7–12 years	15,835	56%	541	52%	16,376	55%
13+ years	4991	18%	128	12%	5119	17%
*Missing*	*1585*	*6%*	*63*	*6%*	*1648*	*6%*
**Marital status**						
Single/Separated/Divorced/Widowed/Other	3525	12%	123	12%	3648	12%
Married/Cohabitating	24,852	87%	917	88%	25,769	87%
*Missing*	*99*	*0%*	*0*	*0%*	*99*	*0%*
**BMI groups**						
Underweight	147	1%	6	1%	153	1%
Normal weight	7950	28%	265	25%	8215	28%
Overweight	11,128	39%	355	34%	11,483	39%
Obese	4860	17%	197	19%	5057	17%
*Missing*	*4391*	*15%*	*217*	*21%*	*4608*	*16%*
**Obstetric & Labour-Level Factors**						
**Previous Cesarean**						
No	22,624	79%	862	83%	23,486	80%
Yes	5784	20%	177	17%	5961	20%
*Missing*	*68*	*0%*	*1*	*0%*	*69*	*0%*
**Obstetrically High Risk**^**e**^						
No	25,784	91%	837	80%	26,621	90%
Yes	2560	9%	201	19%	2761	9%
*Missing*	*132*	*0%*	*2*	*0%*	*134*	*0%*
**Referred during labor**						
No	21,216	75%	728	70%	21,944	74%
Yes	7252	25%	311	30%	7563	26%
*Missing*	*8*	*0%*	*1*	*0%*	*9*	*0%*
**Gestational Age at Birth**						
24–33	590	2%	36	3%	626	2%
34–36	1907	7%	90	9%	1997	7%
37–42	25,979	91%	914	88%	26,893	91%
**Birthweight (grams)**						
< 1500	249	1%	26	3%	275	1%
1500–2500	2427	9%	119	11%	2546	9%
2500–3500	17,359	61%	587	56%	17,946	61%
3500–4500	8028	28%	279	27%	8307	28%
4500+	308	1%	24	2%	332	1%
*Missing*	*105*	*0%*	*5*	*0%*	*110*	*0%*
**Onset of Labor**						
Spontaneous	24,461	86%	881	85%	25,342	86%
Induced	2957	10%	109	10%	3066	10%
*Missing*	*1058*	*4%*	*50*	*5%*	*1108*	*4%*
**Parity**						
0	15,223	53%	497	2%	15,720	53%
1+	13,194	46%	529	2%	13,723	46%
*Missing*	*59*	*0%*	*14*	*0%*	*73*	*0%*
**Fetal Presentation**						
Cephalic	24,787	87%	866	83%	25,653	87%
Breech	2728	10%	122	12%	2850	10%
Other	904	3%	52	5%	956	3%
*Missing*	*57*	*0%*	*0*	*0%*	*57*	*0%*
**Number of neonates**						
1 neonate	27,520	97%	974	94%	28,494	97%
≥ 2 neonates	956	3%	66	6%	1022	3%
**Available facility-Level Factors**						
**Obstetrician Performed surgery**						
No	11,782	41%	450	43%	12,232	41%
Yes	16,684	59%	590	57%	17,274	59%
*Missing*	*10*	*0%*	*0*	*0%*	*10*	*0%*
**Teaching Facility**						
No	5058	18%	374	36%	5432	18%
Yes	23,418	82%	666	64%	24,084	82%
**Total Deliveries at Facility**						
< 3500	7731	27%	336	32%	8067	27%
[3500,10,000)	12,148	43%	590	57%	12,738	43%
10,000+	7431	26%	103	10%	7534	26%
*Missing*	*1166*	*4%*	*11*	*1%*	*1177*	*4%*
**Urban Facility**						
No	25,433	89%	740	71%	26,173	89%
Yes	2993	11%	294	28%	3287	11%
*Missing*	*50*	*0%*	*6*	*1%*	*56*	*0%*
**Indication for CS**						
Fetal Indication	4997	18%	134	13%	5131	17%
Failure to Progress/Dystocia	7010	25%	131	13%	7141	24%
Maternal or Obstetric Indication	1918	7%	127	12%	2045	7%
Multiples/Malpresentation	2390	8%	88	8%	2478	8%
Elective	3286	12%	41	4%	3327	11%
No Other Indication	8832	31%	517	50%	9349	32%
*Missing*	*43*	*0%*	*2*	*0%*	*45*	*0%*

^a^ Democratic Republic of Congo, Niger, Nigeria

^b^ Cambodia, India, Nicaragua, Paraguay, Phillipines, Thailand, Vietnam, Algeria, Ecuador, Peru, Sri Lanka, Kenya, Nepal, Uganda, China

^c^ Argentina, Japan, Cuba, Brazil, Mexico

^d^ Medically High Risk Definition: is a dichotomous variable whereby women are considered to be medically high risk if they have chronic hypertension, cardiac or renal disease, pulmonary pathology, diabetes, malaria, sickle cell disease, severe anemia, urinary tract infection, severe condylomatous disease, or HIV or a condition associated with HIV

^e^ Obstetrically High Risk Definition: is a dichotomous variable whereby women are considered obstetrically high risk if they experience hypertension in pregnancy, pre-eclampsia, eclampsia, or have suspected fetal growth impairment

*p*-values comparing women experiencing adverse outcomes to those who did not, adjusted for country of birth, are shown in the univariate analysis in Table 4

### Univariate analysis (unadjusted odds ratios, UOR)

Odds ratios (ORs) and corresponding 95% confidence intervals (CIs) from the univariate analysis are presented in the first column of Table 3. The composite maternal adverse outcome of intrapartum cesarean birth was less likely to occur in women who delivered in a medium or high human development index country vs. a low human development index country, overall (*p* = 0.003). It was also less likely to occur in women who had at least four antenatal visits (UOR 0.46 [0.35,0.59], *p* < 0.0001), in babies with a birthweight of 3500–4499 (vs. 2500–3499; UOR 0.75 [0.59,0.97], *p* < 0.0001), and in women with a history of prior cesarean birth (vs. no prior cesarean; UOR 0.66 [0.51,0.86], *p* = 0.002).

Women who were medically high risk (UOR 2.17 [1.70,2.77], *p* < 0.0001), were less educated (0–6 years versus 13 or more years; UOR 1.82 [1.26,2.63], *p* < 0.0001), were obstetrically high risk (UOR 4.48 [3.50,5.74], *p* < 0.0001), were referred in labor (UOR 1.92 [1.47,2.50], *p* < 0.0001), or were preterm (24–33 weeks UOR 5.16 [3.33,8.00] and 34–36 weeks UOR 2.82 [2.06,3.87], *p* < 0.0001) had an increased risk of the adverse outcome. Similarly, women with babies less than 2500 g were at increased risk (< 1500 g UOR 7.37 [4.33,12.6] and 1500–2499 g UOR 2.06 [1.52,2.78], *p* < 0.0001), as were those with multiple gestation (UOR 2.17 [1.44,3.28], *p* = 0.0002). Finally, compared to the elective category of indications, women with a maternal or obstetric indication (UOR 6.81 [4.25,10.9]) or no other indication (UOR 2.41 [1.56,3.73) were at an increased risk of the composite adverse outcome, *p* < 0.0001.

### Multivariable analysis (adjusted odds ratios, AOR)

Odds ratios (ORs) and corresponding 95% confidence intervals (CIs) from the multivariate analysis are presented in the second column of Table 4. The composite maternal adverse outcome of intrapartum cesarean birth was overall less likely to occur in women who delivered in a medium or high human development index country vs. a low human development index country (AOR 0.07[0.01,0.85] and AOR 0.02[0.001,0.39], respectively; *p* = 0.03), women having four or more antenatal visits (AOR 0.60; 95% CI: 0.43–0.84; *p* = 0.003), and women with malpresenting fetuses (vs. cephalic: breech AOR 0.52; CI: 0.31–0.87; *p* = 0.04).

**Table 4:** Odds ratios and adjusted odds ratios with 95% confidence intervals from mixed effects regression models of factors associated with adverse outcomes following intrapartum cesarean birth, WHOGS 2004–2008

	Univariate Analysis			Multivariable Analysis		
	OR	95% CI	***P***-value	AOR	95% CI	***P***-value
**Woman-Level Factors**						
**Human Development Index 2008 (ref: Low**^**a**^**)**			0.003			0.03
Medium^b^	0.05	(0.01, 0.46)		0.07	(0.01, 0.85)	
High^c^	0.01	(< 0.001, 0.16)		0.02	(0.001, 0.39)	
**Antenatal Visits (ref: < 4)**			<.0001			0.003
4+	0.46	(0.35, 0.59)		0.60	(0.43, 0.84)	
**Medically high risk**^**d**^ **(ref: No)**			<.0001			0.0004
Yes	2.17	(1.70, 2.77)		1.81	(1.30, 2.51)	
**Age groups (ref: 19–34)**			0.07			0.30
0–18	1.00	(0.68, 1.46)		0.63	(0.35, 1.14)	
35+	1.39	(1.05, 1.84)		0.93	(0.65, 1.35)	
**Education groups (ref: 13+)**			<.0001			0.01
0–6	1.82	(1.26, 2.63)		1.64	(1.03, 2.63)	
7–12	0.97	(0.70, 1.34)		0.98	(0.66, 1.44)	
**Marital status (ref: Single/Separated/Divorced/Widowed/Other)**			0.79			0.61
Married/Cohabitating	1.05	(0.75, 1.46)		0.88	(0.54, 1.44)	
**BMI groups (ref: [18.5,25))**			0.16			0.10
[0,18.5)	2.47	(0.66, 9.18)		4.66	(1.12, 19.4)	
[25,30)	1.17	(0.87, 1.56)		1.33	(0.95, 1.86)	
30+	1.42	(0.99, 2.04)		1.29	(0.84, 1.96)	
**Obstetric & Labour-Level Factors**						
**Obstetrically High Risk**^**e**^ **(ref: No)**			<.0001			<.0001
Yes	4.48	(3.50, 5.74)		3.67	(2.58, 5.23)	
**Referred during labor (ref: No)**			<.0001			0.08
Yes	1.92	(1.47, 2.50)		1.37	(0.96, 1.96)	
**Gestational Age at Birth (ref: 37–42)**			<.0001			0.13
24–33	5.16	(3.33, 8.00)		2.24	(0.96, 5.25)	
34–36	2.82	(2.06, 3.87)		1.36	(0.82, 2.28)	
**Birthweight (grams) (ref: [2500,3500))**			<.0001			0.14
< 1500	7.37	(4.33, 12.6)		1.64	(0.54, 4.97)	
[1500,2500)	2.06	(1.52, 2.78)		0.97	(0.61, 1.54)	
[3500–4500)	0.75	(0.59, 0.97)		0.72	(0.51, 1.00)	
4500+	1.70	(0.83, 3.47)		1.68	(0.68, 4.12)	
**Onset of Labor (ref: Spontaneous)**			0.71			0.68
Induced	1.07	(0.75, 1.53)		0.91	(0.59, 1.42)	
**Parity (ref: 0)**			0.92			0.25
1+	1.01	(0.83, 1.24)		1.21	(0.88, 1.68)	
**Fetal Presentation (ref: Cephalic)**			0.15			0.04
Breech	0.72	(0.52, 1.01)		0.52	(0.31, 0.87)	
Other	0.89	(0.53, 1.50)		0.66	(0.31, 1.39)	
**Number of neonates (ref: 1)**			0.0002			0.24
2+	2.17	(1.44, 3.28)		1.46	(0.78, 2.76)	
**Previous Cesarean (ref: No)**			0.002			0.39
Yes	0.66	(0.51, 0.86)		0.82	(0.53, 1.28)	
**Available facility-Level Factors**						
**Obstetrician Performed surgery (ref: No)**			0.28			0.57
Yes	0.84	(0.61, 1.15)		0.89	(0.59, 1.34)	
**Teaching Facility (ref: No)**			0.08			0.16
Yes	0.37	(0.12, 1.14)		0.33	(0.07, 1.57)	
**Total Deliveries at Facility (ref: < 3500)**			0.27			0.26
[3500,10,000)	2.21	(0.70, 7.01)		2.84	(0.64, 12.6)	
10,000+	3.65	(0.54, 24.8)		5.39	(0.43, 67.0)	
**Urban Facility (ref: No)**			0.16			0.26
Yes	2.49	(0.70, 8.89)		2.89	(0.46, 18.3)	
**Indication CS (ref: Elective)**			<.0001			<.0001
Fetal Indication	1.36	(0.84, 2.20)		1.31	(0.65, 2.64)	
Failure to Progress/Dystocia	1.11	(0.69, 1.79)		1.39	(0.70, 2.76)	
Maternal or Obstetric Indication	6.81	(4.25, 10.9)		4.74	(2.36, 9.50)	
Multiples/Malpresentation	1.50	(0.88, 2.54)		1.65	(0.73, 3.73)	
No Other Indication	2.41	(1.56, 3.73)		1.77	(0.94, 3.33)	

^a^ Democratic Republic of Congo, Niger, Nigeria

^b^ Cambodia, India, Nicaragua, Paraguay, Phillipines, Thailand, Vietnam, Algeria, Ecuador, Peru, Sri Lanka, Kenya, Nepal, Uganda, China

^c^ Argentina, Japan, Cuba, Brazil, Mexico

^d^ Medically High Risk Definition: is a dichotomous variable whereby women are considered to be medically high risk if they have chronic hypertension, cardiac or renal disease, pulmonary pathology, diabetes, malaria, sickle cell disease, severe anemia, urinary tract infection, severe condylomatous disease, or HIV or a condition associated with HIV

^e^ Obstetrically High Risk Definition: is a dichotomous variable whereby women are considered obstetrically high risk if they experience hypertension in pregnancy, pre-eclampsia, eclampsia, or have suspected fetal growth impairment

*p*-values comparing women experiencing adverse outcomes to those who did not, adjusted for country of birth, are shown in the univariate analysis in Table 4

Women who were medically high risk (vs. not medically high risk: AOR 1.81; CI: 1.30–2.51, *p* < 0.0004), had less education (0–6 years) (vs. 13+ years; AOR 1.64; CI: 1.03–2.63; *p* = 0.01), were obstetrically high risk (vs. not high risk; AOR 3.67; CI: 2.58–5.23; *p* < 0.0001), or had a maternal or obstetric indication or no other indication (vs. elective: AOR 4.74; CI: 2.36–9.50; *p* < 0.0001) had increased odds of the adverse outcome.

## Discussion

In this analysis of 29,516 women who underwent intrapartum cesarean birth in 22 countries in Africa, Asia, and Latin America, factors independently associated with the composite adverse maternal outcome following intrapartum cesarean included women being medically or obstetrically high risk or having a maternal, obstetric, or no other indication for cesarean birth. Factors associated with a reduction in the composite outcome were giving birth in a country of medium or high Human Development Index of the country in which the woman delivered, having had at least four antenatal care visits, and having a breech or other malpresenting fetus compared to cephalic. We hypothesize that our result related to malpresentation represents the fact that most women with non-cephalic fetuses were likely delivered by pre-labor cesarean birth, and only those with very advanced, spontaneous labor delivered vaginally with overall good outcomes.

A potential target for modifying risk associated with intrapartum cesarean birth is attendance at antenatal care. Our analysis suggests that four or more antenatal care visits during pregnancy is associated with 40% fewer adverse outcomes in women who experienced an intrapartum cesarean birth. Though antenatal attendance has previously shown an association with cesarean birth, we could not find other analyses suggesting that increased antenatal care attendance reduces adverse maternal outcomes of intrapartum cesarean birth [18]. This suggests the possibility that this variable is confounded by another variable or may reflect that women with preterm birth attend less antenatal visits. However, researchers in the United States have suggested that improving recovery after cesarean birth does begin in early antenatal care [19]. WHO has recently published recommendations that women have at least eight antenatal contacts during the course of pregnancy to improve outcomes, which might have an impact on this composite outcome, but we have no evidence of this potential effect [20, 21].

Improving maternal outcomes of intrapartum cesarean birth could involve triaging women during antepartum care to assess their need for specialized care as another potential strategy to improve outcomes. Women with obstetric complications of pregnancy (i.e. hypertensive disorders or suspected fetal growth impairment), who were shown to have an increased risk of maternal adverse outcomes, might benefit from additional prenatal management, a more skilled antenatal care provider, or management by a high-risk service in labor [22]. Specialty clinics and risk scoring have been previously explored as methods to meet the needs of subpopulations of women with special antepartum needs [23, 24]. As such, we hypothesize that optimizing management or prevention of these obstetric and medical issues, and recommending specific birth planning that takes current or potential complications into account, may improve maternal outcomes in the event an intrapartum cesarean birth occurs.

Our definition of obstetrically high risk included women with hypertension in pregnancy, pre-eclampsia, eclampsia, or have suspected fetal growth impairment, with other high risk variables (history of cesarean birth, number of gestations, fetal presentation) entered separately into the model. The WHO, in addition to a number of other organizations such as the American College of Obstetricians and Gynecologists, have published guidelines for managing hypertension in pregnancy as hypertensive disorders are a contributor to maternal mortality [25–28]. Additionally, women referred during the course of labor also had an increased risk of the composite adverse outcome. WHO, in its guidelines on respectful maternity care during labor and childbirth, specifically notes that delivery of high-quality care requires timely and appropriate referral in labor when complications are encountered, through improved infrastructure and established referral pathways [20, 29, 30].

Finally, our analysis found that among women with a maternal or obstetric indication for intrapartum cesarean birth, as compared to cases where the indication was reported as “elective” (see Table 1 for definition), women were more likely to experience the composite adverse outcome. Compared to these cases, women that experience emergency cesarean birth have been shown to have worse maternal and fetal/neonatal outcomes [31, 32]. That being said, cesarean birth should be provided at the right time, for appropriate reasons, and with high-quality technique [7]. It is a major abdominal surgery that can in itself result in adverse outcomes, and can be quite expensive to deliver, so it should only be provided when medically necessary [1–3, 33, 34]. Indication for cesarean birth is a modifiable risk factor in that it can be modified to promote best obstetric practices. Guidelines for prevention of primary cesarean birth have been produced to assist in the decision-making process to proceed to cesarean birth [35, 36].

The limitations of this study include the fact that the data were collected 10 years ago and that unmeasured facility, sociodemographic, or obstetric variables may confound the results. Due to the large amount of missingness for some maternal outcome variables (intrapartum and postpartum blood transfusion, internal iliac artery ligation, and postpartum urogenital fistula) they were not included in the definition of the composite outcome, although we ideally would have wanted to include them. Additionally, 16% of BMI data was missing, and while the weight parameter was meant to reflect the most recently recorded weight of a woman, the time of collection could vary. We also note that the criteria for definition of the various morbidities was according to local practices and the methodology of the survey did not impose any specific definition, which makes interpretation of some the results more challenging. For example, the collecting agency did not specify criteria for ICU admittance. We noted in our initial analysis that the component of the composite outcome that accounted for the most adverse outcomes was ICU admission. This was a subjective parameter in the data set as each hospital had its own admittance criteria. We ran a histogram of days in the ICU and found that most women were only admitted to the ICU for 1 day (data not shown); we hypothesized this was the result of it being common practice in some settings where the only hospital location capable of monitoring a post-operative patient is the ICU. In order to try to refine the definition to include only those women who had severe morbidity and were not just in the ICU for monitoring, we adjusted the definition to stays of greater than or equal to 2 days.

A previous paper observed the association of cesarean birth, considering indication, with maternal and perinatal outcomes in Asian populations from this dataset [10]. Our analysis adds to this prior analysis by evaluating the association of additional, potentially modifiable risk factors with a composite adverse maternal outcome following intrapartum cesarean birth in the entire dataset. Other strengths of this analysis are the use of a large data set, the collection of multiple variables potentially associated with the outcome, the fact that the survey was designed to assess method of birth, and that the multi-country data were collected using a standard approach/protocol/measurement tool and abstracted from routine medical records [9].

## Conclusion

We found reduced adverse maternal outcomes following intrapartum cesarean birth associated with women giving birth in medium or high human development index countries and those who attended ≥ 4 antenatal visits. Maternal adverse outcomes of intrapartum cesarean birth were increased in medically and obstetrically high risk women and those with a maternal or obstetric indication for cesarean birth.

## Data Availability

Requests for data should be made to the WHO Human Reproductiuon Program for consideration.

## Abbreviations

WHO: World Health Organization
WHOGS: WHO Global Survey of Maternal and Perinatal Health
UOR: Unadjusted odds ratio
AOR: Adjusted odds ratios
